# Pharmacosomes: An Emerging Novel Vesicular Drug Delivery System for Poorly Soluble Synthetic and Herbal Drugs

**DOI:** 10.1155/2013/348186

**Published:** 2013-09-09

**Authors:** Archana Pandita, Pooja Sharma

**Affiliations:** Nanomedical Research Centre, I.S.F. College of Pharmacy, Ferozepur G.T. Road Ghal Kalan, Moga, Punjab 142001, India

## Abstract

In the arena of solubility enhancement, several problems are encountered. A novel approach based on lipid drug delivery system has evolved, pharmacosomes. Pharmacosomes are colloidal, nanometric size micelles, vesicles or may be in the form of hexagonal assembly of colloidal drug dispersions attached covalently to the phospholipid. They act as befitting carrier for delivery of drugs quite precisely owing to their unique properties like small size, amphiphilicity, active drug loading, high entrapment efficiency, and stability. They help in controlled release of drug at the site of action as well as in reduction in cost of therapy, drug leakage and toxicity, increased bioavailability of poorly soluble drugs, and restorative effects. There has been advancement in the scope of this delivery system for a number of drugs used for inflammation, heart diseases, cancer, and protein delivery along with a large number of herbal drugs. Hence, pharmacosomes open new challenges and opportunities for improved novel vesicular drug delivery system.

## 1. Introduction

The novel drug delivery system has been exploited a lot in the past few decades, and attention is also being paid to further develop this system. The two ideal requirements for a system to be novel are

1. drug delivery at a predetermined rate and for pre-determined span of time;
2. conveying the active entity to the target site.

**Figure 1:**
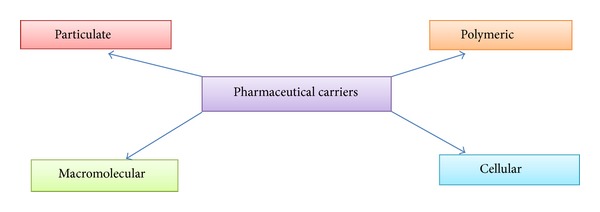
Different types of pharmaceutical carriers.

At this point of time, there is no such system that can fulfil all these requirements. So a lot of efforts are required to accomplish them using novel approaches. These goals are being achieved by concentrating attention either on drug distribution by unifying drug into a carrier system, modifying molecular drug construct, or by restraining drug release in the bioenvironment to ascertain assigned distribution profile. Novel drug delivery attempts to minimize the side effects and maintain relatively unvarying and potent levels of drug in the body. The carriers or chemical derivatization may help to localize the drug action spatially in diseased tissue or organ or adjacent to it [1]. Among the different pharmaceutical carriers (Figure 1), the vesicular carriers are extremely organized assemblies of bilayers of lipid that may be single or concentric in nature formed when the building blocks (amphipathic) of these bilayers encounter water [2]. The clinical use of many drugs particularly chemotherapeutic agents is limited because of their narrow therapeutic window [1]. So the novel approaches in drug delivery serve as a helping hand to achieve all these imperative goals.

The advantages of novel drug delivery system includes [2]

1. incorporation of therapeutic dose at controlled rate,
2. sustaining drug concentration within an optimal range,
3. optimum dose at right time and at right location,
4. relationship between maximum efficacy and the dose of drug,
5. minimizes adverse or toxic effects,
6. freedom from frequent dose intake,
7. improved patient compliance.

## 2. Vesicular Drug Delivery System

Vesicular drug delivery system is one of the systems that can improve the bioavailability of the drug and the reduction in toxicity by drug targeting to the specific site. Bingham pioneered the biologic origin of vesicular systems in 1965, and hence named them Bingham bodies [3]. As a consequence, a large account of vesicular systems like liposome [4], noisome [5], and pharmacosome [6] came into existence. As the drug reaches directly to the site of action, there is a reduction in drug toxicity and no conflicting effects are seen. 

## 3. Advantages of Vesicular System [7]

1. It is an efficient method for reducing the drug toxicity and targeting it to the site of action.
2. It is capable of consolidating hydrophilic and hydrophobic drugs.
3. It helps to reduce the expense of treatment.
4. It improved bioavailability of poorly soluble drugs.
5. It sustained release by receding the time of drug elimination of rapidly metabolizable drugs.
6. It overcomes the complications regarding stability, solubility and degradation of the drug.
7. It acts as drug reservoir by encapsulating the drug and overcoming the problems of conventional dosage forms.
8. These carriers correspond to the structure and function of biomolecules and hence are biocompatible and biodegradable.

**Figure 2:**
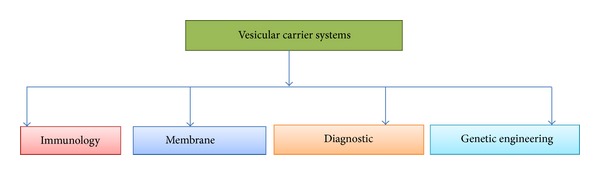
Applications of vesicular carrier systems.

The properties of the vesicle depend on its varying configuration, composition, shape, entrapment, apparent charge and concentration. “The vesicular carrier systems have observed a number of applications in various fields (Figure 2)”. All those preparations that load the drugs passively like microemulsions or carriers that are temperature, pH or magnetically sensitive, consequently lead to less drug loading and leakage of the drug in formulation [8–11]. 

**Figure 3:**
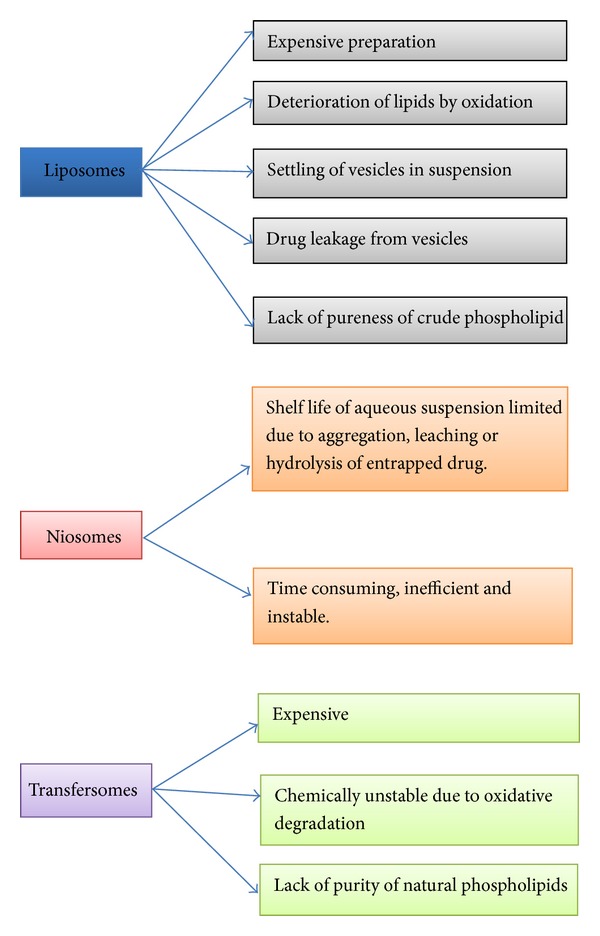
Complications with various vesicular systems.

Some vesicular system associated problems are shown in Figure 3 [2, 12]. 

The major complications that arise with most of the drugs are their poor solubility that results in poor bioavailability because the rate of absorption as well as the extent of drug absorbed depends on solubility of the drug. For this purpose, Gordon Amidon and his collaborators, in 1995, introduced a classification system for the drugs based on their solubility and permeability, called the Biopharmaceutics Classification System (BCS) [13]. There are four classes according to this system: Class I drugs substances showed high permeability and high solubility, Class II drugs showed high permeability and low solubility, Class III drugs exhibited low permeability and high solubility, whereas Class IV drugs showed low permeability and low solubility. So dissolution was a limiting factor of Class II and Class IV drugs according to BCS system [14]. The rate of drug release depends on the intrinsic solubility which in turn relies on the size of particles, structural order of the drug, derivatized form of the drug, and so forth. So a number of concepts have been applied to enhance the solubility of such poorly soluble drugs like size reduction, solubilising in a surfactant, forming drug derivative that is water-soluble, conversion of liquid medications into dry free flowing powders, changing solid state of the drug, and forming solid dispersion and complexes with agents, for example, metals, cyclodextrin [15]. The use of lipids as carrier based systems has recently shown to improve the solubility of drugs that are poorly soluble in water as the increase in solubility will result in enhanced bioavailability. The lipid nanoparticles have the property to adhere like the gastrointestinal tract and once they adhere, they can release the drug at appropriate site of action. Solid lipid nanoparticles and nanolipid carrier systems have been used for the purpose [16, 17]. Another technique for improving solubility using complexation of the drug with phospholipids has demonstrated enhancement in absorption along with the permeation of lipid-complexed drug molecule [11, 18]. Development in the field of vesicular system has led to drug targeting, sustained release, and reduction in toxicity of the drugs [19]. Owing to the advantages associated with the pharmacosomes, it will be a landmark in the field of vesicular drug delivery system. 

## 4. Pharmacosomes

Pharmacosome may be defined as a neutral molecule possessing both positive and negative charge, water-loving and fat-loving properties, and an optimum ratio of polyphenol with phospholipids in a complex form. The drugs are present in a dispersion form in these lipoidal drug delivery system conjugated by electron pair sharings and electrostatic forces or by forming a hydrogen bond with lipids [20]. Pharmacosome is derived from the word “Pharmakon” which means drug and “soma” meaning carrier. It means a vesicular system in which the drug is associated with the carrier. These lipid conjugated vesicles may exist as colloidal, nanometric size micelles, vesicles or may be in the form of hexagonal assembly enjoying a functional hydrogen atom banking upon the architecture of the complex. The drug molecule with a free carboxylic or functional hydrogen atom like amino, hydroxyl groups, is converted to an ester with the help of the hydroxyl moiety of the lipid, resulting in the formation of a prodrug. A spacer chain may or may not be used for this purpose. The prodrug possesses both hydrophilic and lipophilic properties. Despite these properties, prodrugs have the capability to reduce interfacial tension, increase the area of contact, and hence improve bioavailability. They aid the deportation through the cell membrane, cell wall, and tissues. If the concentration is increased beyond a level, it may exist in an intermediate state between liquid and crystal [21]. On contact with water, these prodrugs assemble into a single or multiple layers resulting in the formation of pharmacosomes. This system is developed by keeping the surface properties as well as the bulk properties of the drug-lipid conjugate in consideration [11]. 

### 4.1. Salient Features of Pharmacosomes [31]

- The physical and chemical traits of the conjugate control the stability of the whole system.
- As they consist of both water-loving and fat-loving properties, they have an ease of passing through the cell membrane, walls, or tissues either by the action of endocytosis or exocytosis.
- The rate of degradation relies on size, nature of functional group present in the drug molecule, fatty acid chain length in lipids, presence, or absence of spacer. All these factors can be varied to optimize *in vivo* pharmacokinetic behaviour.
- They can be administered via topical, oral, extra- or intravascular route.

### 4.2. Materials for Pharmacosomes [21, 32, 33]

The following components may be utilised for the preparation of pharmacosomes (Table 1).

## 5. Preparation of Pharmacosomes

Initially for the formation of pharmacosomes, there is a need of drug-lipid conjugate. For this purpose, the salt form of the drug is converted into the acidic form to expose the functional hydrogen atom to form a complex. The aqueous solution of the drug is acidified, extracted using chloroform, and subsequently recrystallised. Then equimolar phospholipid concentration is taken and dissolved in an organic solvent, which is then evaporated under vacuum at a definite temperature. The complex is then collected as a dry residue after placing it in a dessicator overnight.

Generally, two methods are followed for the preparation of pharmacosomes. They areas follows.

### 5.1. Solvent Evaporation Technique

- Hand-shaking method: In this technique, the drug-lipid conjugate is mixed with an organic solvent, which under the conditions of vacuum deposits a thin film on the walls of round-bottom flask and yields a vesicular suspension when hydrated with aqueous medium.
- Rotary evaporator.

### 5.2. Ether-Injection Technique

In this technique, the drug-lipid complex is dissolved in an organic solvent. This mixture is then slowly injected into a heated aqueous agent, resulting in the formation of vesicles. The state of amphiphiles depends on the concentration. When the concentration is less, amphiphiles introduce a monomer state but as the concentration is increased, variety of structures may be formed, that is, round, cylindrical, disc, cubic, or hexagon type. Mantelli and collaborators studied the comparative effect of prodrug of diglyceride with a standard surfactant, dodecylamine hydrochloride on the interfacial tension. It was concluded that above the critical micellar concentration, long cylinders were observed in hexagonal arrangement, and prodrug exhibited liquid-crystalline phase, exhibiting large molecular structures [11, 22, 23].

### 5.3. Other Approaches

- Another approach for producing pharmacosomes is to incorporate a hydrophobic drug into a polymer fabricated from a glycol and aspartic acid derivative resulting in formation of a biodegradable micelle drug conjunct. Being a water soluble monomeric conjunct of drug, the chances of precipitation of drug on dilution are reduced [11, 34].
- A modified technique involving diluted lyotropic liquid crystals of amphiphilic drugs was used by Goymann and collaborators for developing fenoprofen drug based pharmacosomes [11, 35].

Despite these conjugations, drug can also be marked to glyceride-containing groups, where the amphiphilic molecules can be dispersed spontaneously [11, 36]. Singh and Jain formulated “vesicular constructs” with the help of stoichiometric concentrations of phosphatidylethanolamine along with phosphatidylcholine and small amount of cholesterol to encapsulate antibiotic amoxicillin in aqueous domain which significantly enhanced cytoprotection [37].

Li and colleagues [38] studied the physicochemical properties of puerarin and its lipid conjugate prepared by traditional method like solvent evaporation, freeze thawing, and particle size reduction technique along with technology of super-critical fluid. Puerarin is an isoflavone used in conditions like high temperature, aches, diabetes, heart disease, respiratory infections like measles, and bowel disturbances like diarrhoea. Initially the conjugate was prepared by adding solution of both phospholipid and drug in the reaction vessel at a pressure of 100 bar and a temperature maintained at 38°C, which was then left for approximately 3 hours. For the complete removal of residual solvent, the flow of carbon dioxide was regulated at 25 mL/min. The complexes prepared by using the SCF technology demonstrated more rapid dissolution and better particle size and morphology.

## 6. Characterisation of Pharmacosomes [2, 11, 21, 32, 33, 39]

### 6.1. Complex Determination

 With the help of FTIR spectrum, the formation of the complex or the conjugate can be determined by correlating spectrum observed in complex sample with that of discrete constituents and also with their mixture.

### 6.2. Stability of Pharmacosomes

Correlating the spectrum of complex at various points of time in the solid state with spectrum of a dispersion in water consisting of small particles, once the product has been lyophilized, is used to evaluate the stability of the system.

### 6.3. Scanning Electron Microscopy/Transmission Electron Microscopy

These techniques can be utilised for studying the surface order of pharmacosomes. The purity grades of the lipid being used and few variables observed during operation (method of preparation, vacuum assigned, and rotational speed) alter the shape and size of pharmacosomes. Pharmacosomes are formed of greasy nature if prepared using lower purity grades of lipids resulting in large aggregate formation and those fabricated using lipids of more than 90% purity grade show susceptibility to degradation due to oxidation, which affects complex stability. So, 80% purity grade is the commonly used phospholipid grade.

### 6.4. Solubility

The modification in solubility caused by complexation can be evaluated using shake-flask technique. In this technique, the organic phase, that is, 1-octanol and aqueous phase, that is, buffer solution at appropriate pH consisting of drug-phospholipid conjugate are consorted, and after constant shaking, equilibrium is maintained at a temperature of 37°C for 1 day. The aqueous phase is separated and then concentration is determined using UV or HPLC technique.

### 6.5. Drug-Lipid Compatibility

Differential scanning calorimetry is a thermoanalytical technique utilised to determine drug-lipid compatibility and their interactions, if any. The thermal response is studied using separate samples and heating them in a sample pan which is closed. The nitrogen gas is purged, and the temperature is maintained in a definite range with a specific heating rate.

### 6.6. Crystalline State Measurement

The crystalline nature of drug can be determined using X-ray diffraction technique. The tube voltages and tube current can be regulated in the X-ray generator. Copper lines may be used as the source of radiation. The scan angle can be regulated. The overall combined intensity of all reflection peaks is projected by area under curve of X-ray powder diffraction pattern that specifies the specimen attributes.

### 6.7. Dissolution Studies

Dissolution studies, *in vitro* are done using various models available for the purpose. The results are assessed on the basis of apprehended activity of the active constituents therapeutically.

## 7. Advantages of Pharmacosomes [11, 40, 41]

- Membrane fluidity does not control the release rate, as the drug is covalently bound. The release rate is affected by the transition temperature of conjugate.
- The release of the drug from pharmacosomes is hydrolysis based, which includes enzymatic hydrolysis also. After absorption, their rate of degradation relies on the size, nature of functional group, fatty acid chain length, and presence or absence of spacer chain.
- As the carrier is covalently bound, there are no chances of drug leakage.
- Being amphiphilic in nature, it can incorporate hydrophilic as well as lipophilic drugs.
- Entrapment efficiency is quite high and predetermined because of covalent linkage of drug and carrier. It remains unaffected by the volume of inclusion.
- Unlike liposomes, there is no need to remove the unentrapped drug.
- There is a reduction in transfer/exchange of phospholipids, and low solubility is envisaged by high density lipoproteins.
- The bioavailability of drugs that are poorly soluble is improved.
- They targeted delivery to the site of infection.
- They reduced cost of therapy.
- Reduction in adverse effects and toxicity.

### 7.1. Advantages of Pharmacosomes over Other Vesicular Systems [11, 32, 33, 42, 43]

- They are less tedious and time consuming than liposomes.
- The process of drug release is hydrolysis rather than bilayer diffusion, surface desorption, or degradation as in case of liposomes.
- Unlike liposomes, the entrapment efficiency of pharmacosomes remain unaffected by the volume of inclusion.
- The membrane fluidity of pharmacosomes is dependent on conjugate phase transition temperature and does not affect the release rate of pharmacosomes due to covalent binding of drug and lipid. However, in case of liposomes, drug release and system stability are governed by membrane fluidity, which in turn is dependent on lipid composition.
- There is no drug leakage or sedimentation due to covalent binding of the drug to the carrier.

## 8. Limitations of Pharmacosomes [12]

- A compound can be synthesised depending on the amphiphilic nature.
- They require superficial as well as mass drug-lipid interaction.
- Covalent type of bond is required to restrict drug leakage.
- Pharmacosomes are susceptible to get fused, aggregate, or hydrolyse by chemicals on storage.

## 9. Applications of Pharmacosomes

- Pharmacosomes demonstrate a wider stability profile and greater shelf life.
- Pharmacosomes have the capacity to augment drug absorption and its transport. Using response surface design, Yue et al. [44] and colleagues optimised the formulated geniposide pharmacosomes and examined their attributes. The ratio of phospholipid to drug, temperature of reaction mixture and concentration of drug were found to be 3, 50°C and 5.5 mg/mL, respectively.
- Pharmacosomes can improve the rate of permeation by improving the membrane fluidity. The transition temperature of vesicles in the form of vesicles and micelles might pose an evident effect on vesicular interaction with biomembrane, hence improving the transfer of drug across membrane.
- Khare [45] demonstrated the prominent effect of cascade fusion system of pharmacosomes at appropriate temperature on drug targeting in an organism by applying heating and cooling phenomenon on tissues.
- Pharmacosomes have achieved a new level by enhancing therapeutic effects of several drugs (Table 2) like pindolol derivative, taxol, bupranolol acid derivative, cytarabin, amoxicillin, dermatan sulphate, and so forth [31, 44, 46].
- Pharmacosomes, the amphiphilic lipid vesicular system, can be used for the development of novel ophthalmic dosage forms. Amphiphilic prodrug forms pharmacosomes, when diluted with tear [47], and modify corneal drug transport and release profile [48].
- Pharmacosomes have greater degree of selectivity for action on specific target cells. Raikhman et al. [49] described pharmacosomes as building particles capable in the transport of biologically active substances including nucleic acids and proteins.
- Semalty and colleagues [21] studied the development of pharmacosomes of aceclofenac and evaluated them. A higher drug content was 91.88% (w/w) for 1 : 1 aceclofenac phospholipid complex and 89.03% (w/w) for 2 : 1 aceclofenac phospholipid complex. The solubility was higher in case of aceclofenac pharmacosomes than aceclofenac. Moreover, the drug release over 4 hrs of dissolution study was only 68.69% in case of free aceclofenac, while it was 79.78% for 1 : 1 aceclofenac pharmacosome and 76.17% for 2 : 1 aceclofenac pharmacosomes for the same span of time.
- Semalty et al. [50] studied the development of diclofenac pharmacosome, and it was found that solubility was enhanced in pharmacosomes (22.1 *μ*g/mL) as compared to diclofenac (10.5 *μ*g/mL). Drug release was also improved from 60.4% of diclofenac to 87.8% of diclofenac pharmacosomes after 10 hrs of dissolution study. Observed drug content of diclofenac pharmacosomes was 96.2 ± 1%.
- Han and colleagues [51] optimized the preparation of 20(S)-protopanaxadiol pharmacosomes and observed the encapsulation efficiency of pharmacosome, which was 80.84 ± 0.53 for a diameter of 100.1 nm and 72.76 ± 0.63 for the diameter of 117.3 nm.
- Ping et al. [41] prepared didanosine pharmacosomes using tetrahydrofuran injection method and studied the *in vivo* behaviour in rats. It was found that pharmacosomes may be a potential delivery system for prolonged effects in targeted tissues and liver targeting.
- Zhang et al. [52], using central composite design, regulated pharmacosomes of 3′,5′-dioctanoyl-5-fluoro-2′-deoxyuridine and observed good targeting efficiency of pharmacosomes *in vivo* and improved drug potential to pass through blood brain barrier.
- Yi-Guang et al. [53] prepared acyclovir pharmacosomes and observed that the plasma proteins in blood absorbed pharmacosomes and interfered with the interactions of erythrocytes and hence reduced haemolytic reaction.
- Semalty et al. [33] prepared aspirin-phospholipid complex (1 : 1 molar ratio) and observed the enhanced bioavailability of aspirin and reduced gastrointestinal toxicity.

## 10. Phytosomes: A Novel Drug Delivery System for Herbal Drugs

Pharmacosomes are also commonly known as phytosomes consisting of drug-phospholipid complexes (see Supplementary Material available online at http://dx.doi.org/10.1155/2013/348186, Figure  S1) and having herbal active ingredient [54, 55].

### 10.1. Properties of Phytosomes [56–60]

1. Phytosomes may be defined as a conglomerate of a herbal drug and lipids like soya lecithin that are developed using stoichiometric ratios of lipid and the herbal constituent in a particular solvent. The spectroscopic analysis explained the interaction between the polar functional groups of substrate and phosphate as well as ammonium groups of polar head of phospholipid by forming hydrogen bond. Here the phytoconstituent gets anchored with phospholipid polar head. It, therefore, amalgamates with membrane. For example, in case of phosphatidylcholine and catechin complex, there is a formation of hydrogen bond between hydroxyl group in phenols of the flavones moiety and phosphate group of phosphatidylcholine. When the nuclear magnetic studies were performed for the complex and compared with pure precursors, there was no change in fatty acid chain signals. It suggested that the active principle got enclosed into two long chains, aliphatic in nature. This resulted in a lipophilic envelope that shields the polar phase of phospholipid as well as the constituent [57].
2. Based on pharmacokinetic as well as pharmacodynamic trials performed in animals as well as human beings, phytosomes are known to improve the bioavailability and absorption of herbal extracts as compared to noncomplexed ones [58].
3. Phytosomes are lipophilic in nature having a definite melting point, free solubility in nonpolar solvents, and moderate solubility in fats.
4. There are some elementary differences between liposomes and phytosomes; otherwise they also adapt a micellar construct on treating with water.

### 10.2. Merits [61–65]

1. Phytosomes improve the bioavailability and stability profiles by improving drug absorption to the intestinal tract as compared to unbound phytoconstituent as well as forming a stable complex with phospholipids.
2. Phytosomes can be used for liver targeting as they increase the solubility in the bile salts.
3. There is a reduction in drug dose due to better absorption of active phytoconstituents.
4. PC gives a synergistic hepatoprotective effect with phytosomes, besides acting as a carrier.
5. Phytosomes may be used in cosmetic industry due to their improved skin penetration and high lipid profile.

## 11. Applications of Phytosomes [57, 59, 66–70]

The phytosomes may be used for the development of several herbal extract (Table 3) complexes to provide beneficial effects using natural products.

## 12. Patented Technologies Related to Phytosomes

A number of herbal extract complexes have been developed and are being patented (Table 4) to solve the issues that restrict the use of herbal products.

## 13. Conclusion

Vesicular systems are the emerging carrier systems in the pharmaceutical industry. Despite having disadvantages of getting fused, aggregated, they still serve as a vital tool for targeting ans sustained drug release. With the improvement in spacer groups and linkages, further drug fate and biological activity may be modified. Yet greater efforts are required towards exploring the mechanism of action and investigating nonbilayer phases. Hence, pharmacosomes have immense potential in improving the drug delivery in case of both natural and synthetic active constituents. Current research trends include cellular targeting using different approaches like PEGylation, biotinylation, and so forth.

## Supplementary Material

Figure S1 shows various types of covalent binding of drug to the phospholipids i.e- pharmacosomes and phytosomes and their advantages over other vesicular systems.

## Figures and Tables

**Table 1:** Components of pharmacosomes.

Component	Requirement
Drugs	Functional hydrogen atom from amino, carboxyl, or hydroxyl group that can be esterified
Solvents	High purity, volatile, and intermediate polarity
Lipid	Phospholipids-phosphoglyceride or sphingolipids

**Table 2:** Therapeutic applications of drugs incorporated in pharmacosomes [22, 23].

Drugs	Results
Amoxicillin	Enhanced protection of cells treatment of peptic ulcers in male rats
Bupranolol hydrochloride	Augmented lymphatic transport and affect intraocular pressure
Cytarabin	Biological activity was enhanced
Dermatan sulphate	Biological activity was enhanced and improved bioavailabity
Pindolol diglyceride	Plasma concentration improved up to three to five folds
Taxol	Biological activity was enhanced

**Table 3:** Various Phytosomes of different herbal constituents.

Phytosomes	Phytoconstituent complexed	Indication
Silybin phytosome	Silybin	Nutraceutical, hepatoprotective, and antioxidant for skin
Ginkgo phytosome	24% ginkgo flavonoids	Lipolytic, vasokinetic, and slowing aging process
Green Tea phytosome	Epigallocatechin gallate	Provides nutrition, anticancer, and nutraceutical and prevents systemic oxidation
Olive oil	Polyphenols	Prevents oxidation, inflammation, and elevated lipid levels
Grape Seed	Procyanidins	Protects heart, provides nutrition, capillarotropic, and prevents systemic oxidation
Haw thorn phytosome	Flavanoids	Protects heart, and provides nutrition, antihypertensive
Centella phytosome	Terpenoids	Venous disorders, and skin disorders
Echinacea	Echinocytes	Provides nutrition and improves immunity
Ginseng phytosome	Ginsenosides	Provides nutrition and improves immunity
Licorice phytosome	18 *β*-glycyrrhetinic acid	Relieving effect
Boswellia phytosome	Boswellic acid	Reduction in inflammation
Crataegus phytosome	Vitexin-2′′-O-rhamnoside	Antioxidant
Escin phytosome	Escin *β*-sitosterol	Antioedema
Ginkgoselect phytosome	Ginkgo Flavonglycosides, ginkgolides, and bilobalide	Improved blood circulation
*Ginkgo biloba* terpenes	Ginkgolides, bilobalide	Relaxing and calming
Curcumin phytosomes	Curcuminoids	Anti-inflammatory
PA_2_ phytosome	Proanthocyanidin A_2_	Antiageing, protection against UV
Resveratrol phytosome	Resveratrol rhizome	Antioxidant
Sericoside phytosome	Sericoside bark root	Antiageing
Visnadex	Visnadin	Vasokinetic
Leucoselect phytosome	Procyanidolic oligomers	Systemic antioxidant, antidiabetic, cardioprotective
Mirtoselect phytosome	Anthocyanoside	Antioxidant, to manage blood vessels of retina and venous insufficiency
Sabalselect	Alcohol, fatty acid, and sterol groups	Protects heart and prevents enlargement of cancerous as well as noncancerous prostate
Polinacea phytosome	Echinacosides and HMW polysaccharides	Immunomodulator
Lymphaselect phytosome	Extracts of lymphaselect roots and barks	Chronic venous disorders and insufficiency of lower limbs

**Table 4:** Various patents related to phytosomes.

Research	Innovation	Patent number	Reference
Olive extracted from leaves or fruits complexed with phospholipids	Improved bioavailability	EP/1844785	[24]
Ginkgo biloba derivatives for treatment of asthma and allergy	Asthma and allergy treated	EP1813280	[25]
Cosmetic and dermatological composition for the treatment of aging or photodamaged skin	Development of a topical skin treatment comprising a substance that stimulates collagen synthesis.	EP1640041	[26]
Use of thymosin for skin treatment and wound healing	Use of thymosin *β*-4 for the treatment of skin and repair of wound	US/2007/0015698	[27]
Isoflavone characteristics	Solubility, colour, taste, and textural attributes improved.	WO/2004/045541	[28]
A herbal pant extract based antioxidant for circulatory and adiposity issues	Antioxidant, treatment of circulatory problems	EP1214084	[29]
Saponin phospholipid complex along with cosmeceutical and pharmaceutical compositions in them	High lipophilicity, improved bioavailability, pharmaceutical, dermatologic, and cosmetic advantages	EPO283713	[30]
