# Supplementary figures and images for: Pharmacosomes: An Emerging Novel Vesicular Drug Delivery System for Poorly Soluble Synthetic and Herbal Drugs
Source: ISRN Pharm. 2013 Sep 9;2013:348186. doi: 10.1155/2013/348186 (PMC3782844; doi:10.1155/2013/348186)

## Graphical Abstract:

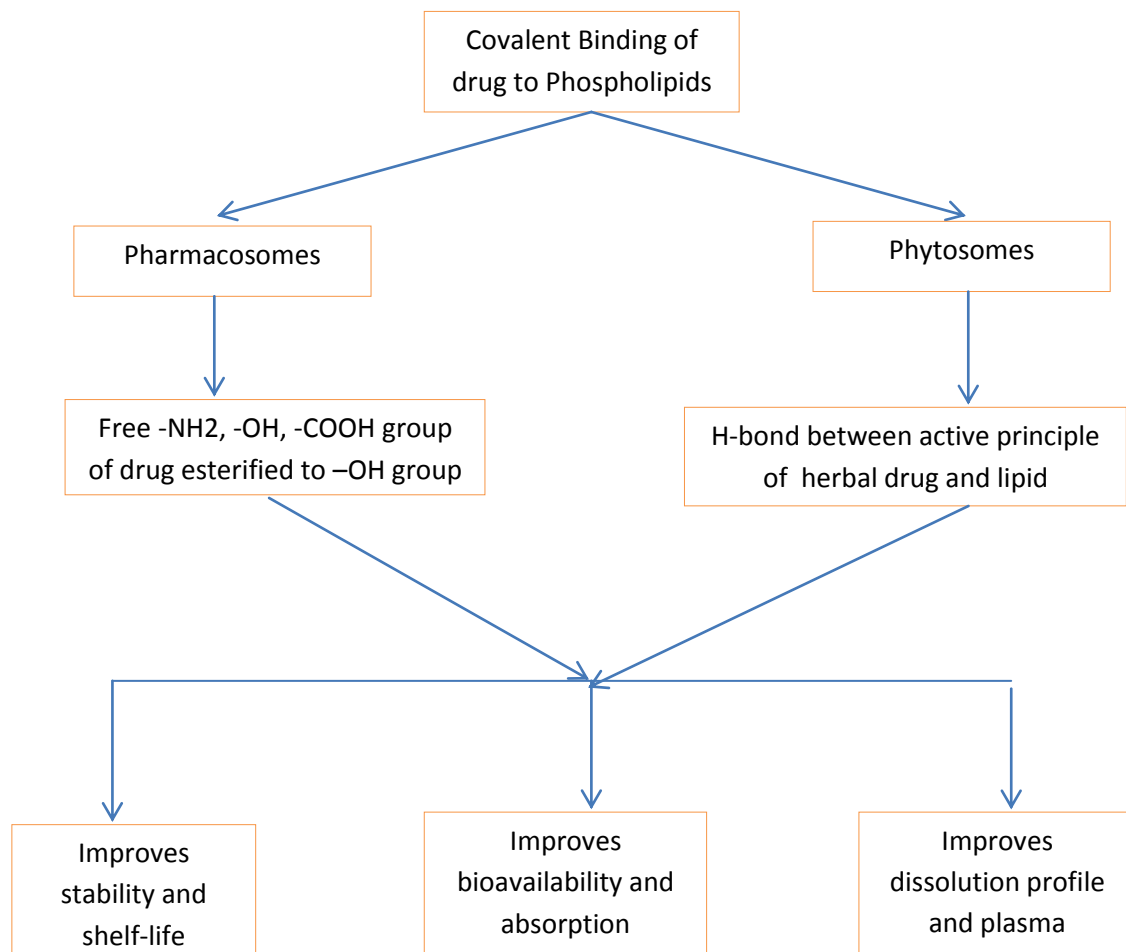

Supplement: Supplementary file 1 — Figure S1 shows various types of covalent binding of drug to the phospholipids i.e- pharmacosomes and phytosomes and their advantages over other vesicular systems. [file 348186.f1.pdf]
